# The Use of Radioactive Iodine in the Treatment of Thyroid Cancer: Novel Aspects

**DOI:** 10.3390/life16101744

**Published:** 2026-10-17

**Authors:** Lambros Athanassiou, Panagiotis Athanassiou, Georgia Kaiafa, Christos Savopoulos, Ifigenia Kostoglou-Athanassiou

**Affiliations:** 1 Department of Rheumatology, St. Paul’s Hospital, GR55134 Thessaloniki, Greece; lambros.ath@gmail.com (L.A.); pathanassiou@yahoo.gr (P.A.); 2 First Department of Propaedeutic Medicine, AHEPA University Hospital, Aristotle University of Thessaloniki, GR54636 Thessaloniki, Greece; gdkaiafa@yahoo.gr (G.K.); chrisavopoulos@gmail.com (C.S.); 3 Department of Endocrinology, Diabetes and Metabolism, Asclepeion Hospital, GR16673 Athens, Greece

**Keywords:** radioactive iodine, thyroid cancer, follicular-derived thyroid carcinoma, dedifferentiation, redifferentiating agents

## Abstract

Thyroid cancer is treated by a combination of surgical treatment in the form of thyroidectomy, administration of radioactive iodine and subsequent administration of thyroxine. The aim of radioactive iodine administration in differentiated thyroid carcinoma patients is remnant ablation, elimination of any microscopic residual disease and treatment of persistent or metastatic disease. The indications for the administration of radioactive iodine have recently changed. In particular, recent guidelines advise against the administration of radioactive iodine in low-risk differentiated follicular-derived thyroid carcinomas. Adjuvant radioactive iodine may be considered in both low–intermediate and intermediate–high risk of recurrence for follicular-derived thyroid carcinomas. Radioactive iodine is recommended in high-risk patients and patients with distant metastatic disease. A small percentage of differentiated follicular-derived thyroid carcinomas become dedifferentiated in the course of the disease, or lose avidity for radioactive iodine, making them resistant to treatment. Recently, it has become apparent that the short-term administration of kinase inhibitors to dedifferentiated thyroid carcinoma patients may restore iodine avidity and enable treatment with radioactive iodine.

## 1. Introduction

Thyroid cancer is common [1,2]. Its increased frequency has been established from autopsy studies, but in clinical practice this was recognized following the identification of nodules by ultrasonography [3,4]. Thyroid nodules in adults are detected by palpation in approximately 5% of cases, by ultrasonography in 30–50% [4,5], and in autopsy material in approximately 45%. Thyroid cancer occurs in large nodules as well as in nodules smaller in diameter than 10 mm, through which they may escape detection by palpation. They are divided into clinically apparent and non-apparent (incidentally discovered) cancers. Thyroid cancer accounts for 90% of all cancers of the endocrine system. It is responsible for 0.5% of all cancer deaths [6]. However, most of the lethality in thyroid cancer patients is related to poorly differentiated, anaplastic thyroid carcinoma and medullary thyroid carcinoma [6,7]. Thyroid cancer incidence has increased over recent years, possibly because of the discovery of thyroid nodules by the extensive use of ultrasonography, the subsequent application of fine needle aspiration biopsy and the use of surgery [8,9]. However, stabilization in the incidence of thyroid cancer and even its decline have been noted recently in the USA, possibly due to the recent trend against overdiagnosis [10,11].

Thyroid cancer is treated by surgery, the use or radioactive iodine treatment and the application of thyroxine [2,12,13]. Relevant surgeries include near-total thyroidectomy or total thyroidectomy. Radioactive iodine treatment is used as indicated in thyroid cancer, specifically in the treatment of papillary and follicular thyroid cancer [14,15]. Thyroxine is administered thereafter.

The concept of treatment for thyroid cancer has been constantly evolving in recent years, both through the treatment of advanced thyroid cancer and in relation to changes in the guidelines for the administration of radioactive iodine [13,14,16,17]. In addition, agents have been introduced for the redifferentiation of dedifferentiated thyroid cancer to reinduce sensitivity to radioactive iodine [17,18,19].

This paper will deal with novel aspects regarding the application of radioactive iodine treatment in thyroid cancer patients with an emphasis on recent advances in the field.

## 2. Methods

All articles published in PubMed regarding radioactive iodine treatment of thyroid cancer in the period from 2000 to 2026 were reviewed. The key words used were thyroid cancer, radioactive iodine treatment, dedifferentiated thyroid carcinoma and dedifferentiating agents. Articles reviewed included clinical trials and reviews. The search was limited to articles in the English language. After elimination of duplicates, eligible articles were read and evaluated.

## 3. Indications for the Administration of Radioactive Iodine to Thyroid Cancer Patients

Radioactive iodine ^131^I is administered in differentiated thyroid carcinoma (papillary and follicular), which accounts for approximately 90% of thyroid cancers [20]. Residual tissue often remains after surgery, or, more rarely, metastases may already be present or may appear later; therapeutic radioactive iodine ^131^I is then administered to kill and destroy the cancer cells [21,22,23,24,25]. The purpose of therapeutic radioactive iodine ^131^I administration is to destroy possible small cancer foci to reduce the risk of tumor recurrence, and to ablate any remaining normal thyroid tissue, thereby facilitating follow-up through thyroglobulin measurement or whole-body radioiodine scintigraphy.

Radioactive iodine was initially administered in the 1930s in the USA for the treatment of hyperthyroidism [26,27]. Almost simultaneously, radioactive iodine treatment was administered to thyroid cancer patients in the USA in New York [28,29]. Beierwaltes’ team in the United States studied the effect of postoperative radioiodine administration in patients with differentiated thyroid carcinoma aged 40 or over 40, and they observed a 20-fold decrease in mortality in patients treated with radioiodine post-surgically as compared to those who only had surgery [30]. In accordance, a positive impact of radioiodine treatment was observed in patients with thyroid cancer of varied histology in a 25-year study in which radioiodine treatment was found to be the most important factor related to survival [31]. Beierwaltes proposed a three-step approach, involving total thyroidectomy followed by thyroid hormone withdrawal and whole-body scintigraphy for the detection of any thyroid remnants and any metastatic disease followed by the administration of radioactive iodine [30]. Thereafter, ablation with radioactive iodine post surgically was established in the treatment of differentiated thyroid carcinoma. Mazzaferri and his colleagues evaluated the effect of radioactive iodine ^131^I treatment on differentiated thyroid carcinoma patients and found a statistically significant difference in recurrence and mortality [31,32]. They observed a decreased recurrence rate across all groups stratified by age and a significant decrease in mortality in patients aged >40 years at diagnosis. They also found decreased cause-specific mortality in patients treated with radioactive iodine, at 3% as opposed to 8% in those not treated with radioiodine [25].

Recommendations [15,33,34] indicate that radioactive iodine should be used in differentiated thyroid carcinoma patients according to the following indications: (a) radioactive iodine administered as adjuvant treatment to destroy possible remnant tumor tissue that might be cancerous in patients categorized as intermediate- and high-risk by histopathology of the surgically resected tumor in order to minimize recurrence risk and (b) treatment of any neoplastic disease present with the aim of managing persistent or recurrent disease in patients with detected metastatic disease. The aims of radioactive iodine treatment include staging of the disease and enabling patient monitoring in the postoperative period. These goals are enabled by inducing non-detectable minimal serum thyroglobulin, absent antibodies against thyroglobulin (TgAb) and negative whole-body scintigraphy, which indicates that there is no residual cancerous thyroid tissue. Adjuvant radioactive iodine treatment also plays additional roles in the management of thyroid carcinoma patients. These include the provision of a curative perspective, prolonging survival related to the thyroid cancer, extension of survival without progression of the disease and reduction in any recurrence risk. Radioactive iodine treatment has a therapeutic role in the management of known thyroid cancer, such as disease staging, postoperative surveillance, extension of survival related to thyroid cancer, prolongation of progression-free time and provision of cure or palliation.

Recently, the American Thyroid Association issued the novel revised management guidelines for patients with differentiated thyroid carcinoma [15], which in fact replaced the guidelines in effect since 2015 [34]. The American Thyroid Association paper focused on the management of differentiated thyroid carcinoma, as compared to the 2015 guidelines which had as their subject both differentiated thyroid carcinoma and thyroid nodules. The 2025 updated guidelines revised the classification of thyroid cancer in four classes, into low-risk, low–intermediate-risk, intermediate–high-risk and high-risk categories. These novel guidelines issued by the American Thyroid Association focused on the identification of patients likely to benefit from radioactive iodine applied as adjuvant treatment in patients, specifically in those in the heterogenous intermediate risk category. The 2025 classification also incorporates histopathologic features not weighted as heavily in earlier guidelines. It takes into account several features, such as microscopically positive margins, multifocality, tumor size, and oncocytic (Hürthle cell) histology, the last of which is now explicitly recognized as being more resistant to radioactive iodine therapy and associated with a higher rate of extra-cervical recurrence. The guideline further emphasizes that stratification should be dynamically viewed; namely, the risk of recurrence should be reassessed in the course of follow-up on the basis of response to the treatment. Thus, stratification of the patient should be performed continuously over the follow-up period.

**Figure 1:**
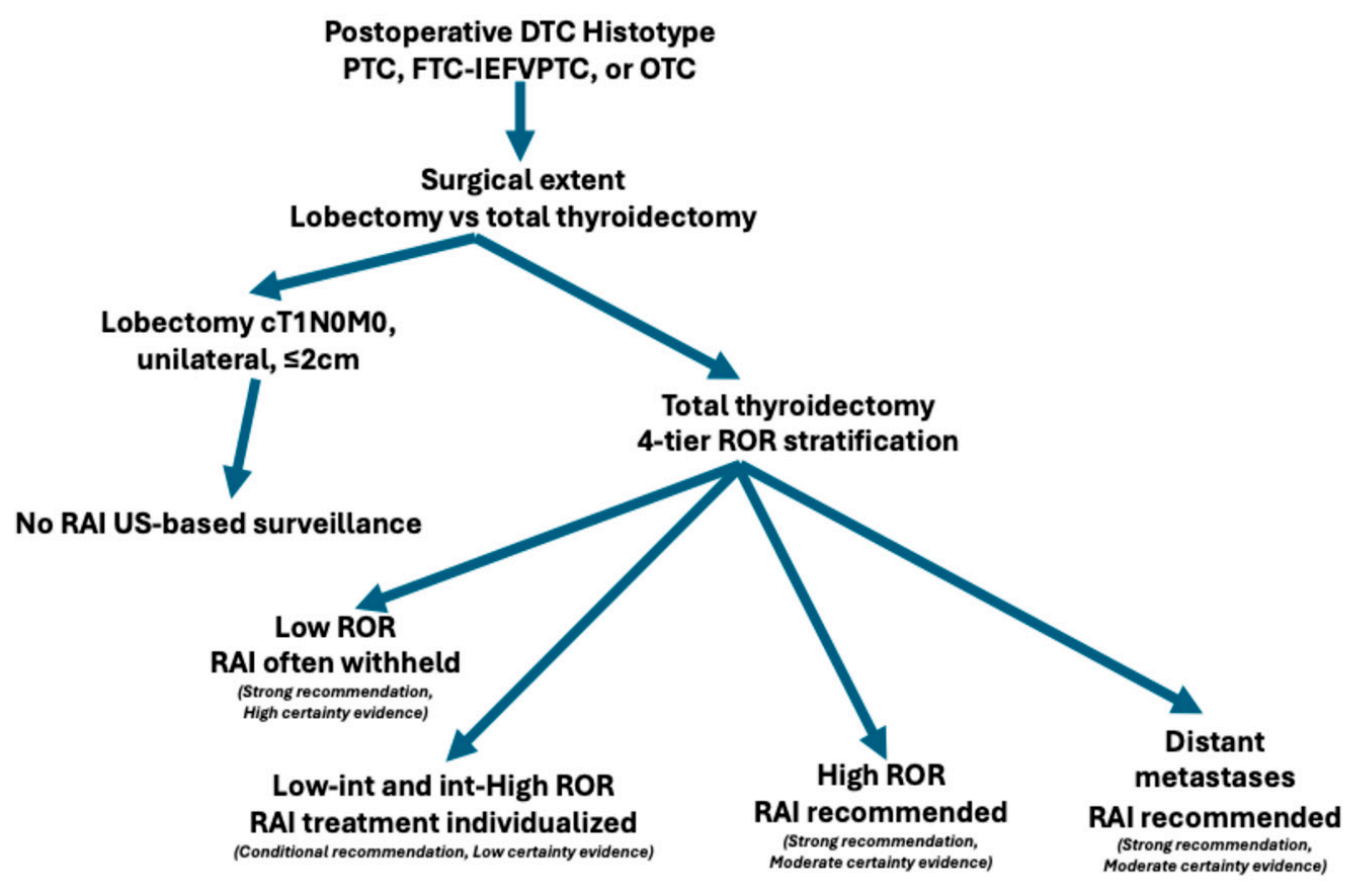
Indications for the treatment of differentiated thyroid carcinoma with radioactive iodine (ATA 2025) [15], (DTC = differentiated thyroid carcinoma, PTC = papillary thyroid carcinoma, FTC = follicular thyroid carcinoma, IEFVTPTC = invasive encapsulated follicular variant of papillary thyroid carcinoma, OTC = oncocytic thyroid cancer, RAI = radioactive iodine, ROR = risk of recurrence).

According to these novel 2025 guidelines for low-risk differentiated thyroid carcinoma patients, routine remnant ablation after total thyroidectomy is not recommended [15]. Adjuvant radioactive iodine may be considered in patients with both low–intermediate- and intermediate–high-risk of recurrence. For high-risk differentiated thyroid carcinoma patients, radioactive iodine treatment following thyroidectomy is recommended (Figure 1). Additionally, in patients with differentiated thyroid carcinoma and distant metastatic disease, radioactive iodine treatment following thyroidectomy is recommended.

The decision to administer radioiodine therapy depends on the treatment goal and is guided by the estimated risk of mortality and the likelihood of persistent or recurrent disease [34,35]. The latest version of the TNM classification (8th version) estimates the risk of mortality from the underlying disease [36]. Unlike the risk of mortality, the risk of recurrence or persistent disease is much higher.

## 4. Differentiated Thyroid Carcinoma, Risk Assessment and Radioiodine Administration

Differentiated thyroid carcinoma patients were broadly categorized into three risk categories, namely low, intermediate and high risk, according to the 2015 American Thyroid Association guidelines [34]. The 2025 American Thyroid Association guidelines now define three separate risk categories based on tumor histotype: one for papillary thyroid cancer and its subtypes, one for follicular thyroid cancer and an invasive encapsulated follicular variant of papillary thyroid carcinoma (IEFVPTC), and one for oncocytic thyroid cancer—each with four risk categories instead of three. The new categories are low-, low–intermediate-, intermediate–high-, and high-risk of recurrence [15,37].

The 2025 American Thyroid Association guidelines [15] substantially revise the risk classification of differentiated thyroid carcinoma by expanding risk stratification to four categories (low, low–intermediate, intermediate–high, high), applying histotype-specific criteria (papillary versus follicular/invasive encapsulated follicular variant of papillary thyroid carcinoma versus oncocytic), and incorporating postoperative biochemical and structural response assessment as a cofactor in the determination of radioactive iodine administration. The low-risk group comprises patients with intrathyroidal disease, absence of adverse histology, absence or presence of minimal vascular invasion, and absent or minimal nodal extension. In this low-risk group, radioactive iodine is not recommended. Carhill et al. investigated the effect of radioactive iodine ablation in patients with low-risk thyroid carcinoma and they showed no impact on survival [38]. Schvartz et al. [37] investigated the effect of adjuvant treatment with radioactive iodine in 1298 low-risk thyroid cancer patients over a period of more than 10 years and they did not find improvement in overall or disease-free survival. Two prospective, randomized, non-inferiority trials investigating the role of radioiodine ablation in differentiated thyroid carcinoma were conducted in Europe. The first, a British study entitled “The IoN Study (Is Ablative Radio-iodine Necessary for Low-Risk Differentiated Thyroid Cancer Patients)”, compared outcomes in low-risk and selected intermediate-risk differentiated thyroid carcinoma patients treated with surgery alone versus those treated with surgery followed by radioactive iodine therapy [39]. The second, the French “Estimabl 2 study (Differentiated Thyroid Cancer: Is There a Need for Radioiodine Ablation in Low-Risk Patients)”, evaluated low-risk differentiated thyroid carcinoma patients who underwent total thyroidectomy (with or without neck dissection). Participants were randomized to receive either 1.1 GBq (i.e., 30 mCi) of ^131^I following stimulation with recombinant human thyroid-stimulating hormone (rhTSH) or no radioactive iodine therapy. The results indicated that radioactive iodine ablation did not provide a significant benefit in low-risk patients [40,41].

The low–intermediate-risk group comprised patients with a single adverse feature such as microscopic extrathyroidal extension, nodal involvement at the level N1a, or unilateral multifocality, or for follicular thyroid carcinoma or oncocytic thyroid cancer, less than four foci of vascular invasion. T3a also falls into this category, as does a microscopically positive posterior surgical margin. In this group the decision to administer radioactive iodine is individualized. Withholding administration of radioactive iodine is favored in the absence of additional adverse indices, especially if thyroglobulin is undetectable or low postoperatively.

The intermediate–high-risk group comprised patients with two or more existing low–intermediate features, such as nodal disease at the N1b level or bilateral multifocality. Clinically evident lateral nodal metastases (cN1b < 3 cm), bilateral multifocality with foci > 1 cm, aggressive histology and vascular invasion are each sufficient criteria for the patient to be categorized as intermediate–high-risk. In this intermediate–high-risk group, the administration of radioactive iodine may be recommended, the activity selected as per anatomic extent of residual disease.

The high-risk group consisted of patients with extrathyroidal extension, distant metastatic disease, incomplete surgical resection or extensive > 4 foci of vascular invasion for follicular or oncocytic carcinoma. Patients with poorly differentiated thyroid carcinoma or high-grade histology, macroscopically incomplete resection, nodal metastases ≥ 3 cm or extranodal extension also belonged to the high-risk group. In this high-risk group the administration of radioactive iodine is recommended; typically, higher activities are preferred.

In conjunction with the risk of recurrence stratification, several factors should be taken into account in the decision to administer radioactive iodine to differentiated thyroid carcinoma patients. These factors include postoperative stimulated or non-stimulated thyroglobulin or anti-thyroglobulin antibody levels (TgAb), functional imaging findings which suggest residual or metastatic disease, and individual patient-level decision factors such as age, comorbidity, and fertility matters.

## 5. Post Radioiodine Treatment Response Assessment

The response criteria classify patients in four parallel groups, each corresponding to subsequent surveillance intensity (Table 1). If the patient has received surgical treatment with total thyroidectomy and radioactive iodine administration, an excellent response is non-stimulated thyroglobulin < 0.2 ng/mL or stimulated thyroglobulin < 1 ng/mL, with negative TgAb and negative imaging findings. If the patient has received only surgical treatment, an excellent response is non-stimulated thyroglobulin levels < 2.5 ng/mL, stable thyroglobulin levels, negative TgAb and negative imaging findings. If the patient has undergone lobectomy, an excellent response is normal or low-risk contralateral lobe (or benign nodules on biopsy) and no abnormal lymph nodes on imaging.

An indeterminate response is marked by the presence of nonspecific findings on imaging, mildly elevated serum thyroglobulin levels, or positive but stable or declining TgAb levels in patients who have been treated with surgery or a combination of surgery and radioactive iodine.

A biochemically incomplete response is identified by elevated serum thyroglobulin concentrations or rising TgAb levels without evidence of structural recurrence in patients treated by surgery or a combination of surgery and radioactive iodine. A structurally incomplete response is identified by evidence of disease recurrence combined with elevated thyroglobulin and/or increased TgAb levels. The presence of clinically persistent disease is marked by biochemical or structural evidence of disease in a period of 90 days following initial treatment. Clinically recurrent disease is marked by biochemical or structural disease identified in patients previously thought of as having an excellent response after treatment. Clinically recurrent disease also presents progression of residual disease that is not clinically or biochemically detectable.

## 6. Radioactive Iodine Activity Selection

Following the decision to administer radioactive iodine activity, selection depends on the intention, namely remnant ablation, adjuvant treatment and treatment of known disease. Once the decision regarding radioactive iodine is taken, activity selection follows the well-established three-intent framework—remnant ablation, adjuvant therapy, and treatment of known disease. The 2025 American Thyroid Association guidelines revised risk stratification criteria substantially but did not introduce new numeric activity thresholds [15]. The administered activity ranges remain consistent with those proposed by the 2015 American Thyroid Association guidelines [34].

For remnant ablation in patients with low risk of recurrence, an activity of 30–50 mCi (1.11–1.85 GBq) is preferred as compared to higher activities based on randomized trials. An activity of 1.1–3.7 GBq (30–100 mCi) is applied for low–intermediate- and intermediate–high-risk thyroid carcinoma patients. An activity of 3.7–5.55 GBq (100–150 mCi) is indicated for high-risk thyroid carcinoma patients, and that of 3.7–7.4 GBq (100–200 mCi), or dosimetry, for distant metastatic disease. Recombinant human TSH (rhTSH) stimulation is preferred over thyroid hormone withdrawal in the low-risk group, given equivalent efficacy and superior quality of life/euthyroid maintenance.

For the administration of radioactive iodine as an adjuvant treatment in patients with intermediate–high risk of recurrence, and in patients with nodal disease N1b, higher nodal burden, multifocality or microscopic extrathyroidal extension, the specific activity should be individualized according to the volume of nodal disease, the number of adverse histologic features, and patient age; activity is typically titrated to lower levels in older age and according to salivary/lacrimal toxicity risk profile. Preparation with rhTSH stimulation is preferred over hormone withdrawal for the administration of radioactive iodine as an adjuvant treatment.

For the treatment of known or persistent disease the recommended activity is stratified by the estimated disease volume. Fixed empiric activity, imaging-adapted activity selection, maximum tolerated activity or lesional dosimetry may be applied. For the treatment of diffuse iodine-avid pulmonary metastatic disease, empiric administration of 3.7–7.4 GBq (100–200 mCi), and 3.7–5.55 GBq in patients over 70, or an activity set by dosimetry, may be applied when calculated to remain below the 80 mCi (2.96 GBq) 48 h whole-body retention threshold and the accompanying limit of 200 cGy to the bone marrow.

## 7. Radioactive Iodine and Mode of Action in Thyroid Cancer

Radioactive iodine is actively transported in thyroid follicular cells as these cells express the sodium-iodide symporter (NIS) [42,43]. Thyroid follicular cells express the sodium-iodide symporter, which is located on the basolateral membrane of these cells [44]. The sodium-iodide symporter, known also as solute carrier family 5 member 5 (SLC5A5), belongs to a family of solute carrier proteins and membrane transport proteins, which have been shown to be involved in the pathophysiology of thyroid cancer. The sodium-iodide symporter actively transports iodide from the blood in the cell against its concentration gradient. The expression of the sodium-iodide symporter is stimulated by TSH binding to the TSH receptor.

Therefore, radioactive iodine is administered following stimulation by TSH, driven either by an increase in TSH levels following thyroid hormone withdrawal or recombinant human TSH/rhTSH administration. Thyroid cancer cells in differentiated thyroid carcinoma express the sodium/iodide symporter, although this expression may be impaired compared to that of normal thyroid cells. Poorly differentiated, anaplastic and radioiodine refractory thyroid carcinoma lose the expression of the sodium/iodide symporter and stop responding to radioactive iodine. Redifferentiation strategies may then be applied to reinstitute the avidity to radioactive iodine, and thus the ability to retain radioactive iodine.

Following the entrance of the iodide within the thyroid cell, it is transported across the apical membrane in the follicular lumen, utilizing pendrin, oxidized by thyroid peroxidase and organifed in tyrosine residues of thyroglobulin. This organification step retains the isotope within the cell or colloid long enough for radiation damage to accumulate.

Radioactive iodine ^131^I decays by the emission of beta particles, with a short tissue penetration range, which cause direct and indirect DNA damage via the induction of double-strand breaks and free radical generation via radiolysis of water that leads to mitotic cell death of the slowly dividing differentiated thyroid cells [45,46]. The effect on the slowly dividing differentiated thyroid cells is the root of the therapeutic effect unfolding slowly over weeks to months. This effect induced by the emitted beta particles is a short path effect and is therapeutically ideal as the damage is concentrated within the thyroid remnant or thyroid tumor and immediately neighboring cells while distant normal tissue is spared. It may still affect nearby non-iodine-avid cells within a radius of a few mm that may still treat microscopic disease with heterogenous expression of sodium-iodide symporter within the tumor. Radioactive iodine ^131^I additionally emits gamma radiation [47,48]. The principal gamma photon, of about 364 keV, has an emission probability of roughly 82%, which is precisely what makes post-therapy imaging possible.

## 8. Clinical Goals of Radioactive Iodine Treatment in Thyroid Cancer

- Remnant ablation post-thyroidectomy which eliminates residual normal thyroid tissue, enabling surveillance and follow-up via serum thyroglobulin and whole-body scintigraphy.
- Application of radioactive iodine as adjuvant therapy for any microscopic residual disease.
- Treatment of existing persistent or metastatic disease, including nodal, pulmonary or osseous metastatic disease which retains the ability to respond to iodine.

## 9. Methods of Radioactive Iodine Administration

The successful therapeutic and diagnostic application of radioactive iodine depends both on its activity and on the adequate preparation of the patient; uptake of the administered radioiodine by residual thyroid tissue or tumor deposits depends on patient preparation.

Adequate elevation of serum TSH upregulates sodium-iodide expression and iodine avidity. Depletion of the body’s stable iodine pool increases the relative uptake of the radioactive tracer. The therapeutic application of radioactive iodine in patients with thyroid cancer should be performed after several practical and safety considerations are taken into account, including pregnancy exclusion for premenopausal female patients, estimation of renal function and radiation protection.

Uptake of ^131^I by thyroid follicular and thyroid cancer cells is mediated by the sodium-iodide symporter, whose expression is under direct transcriptional control of TSH acting via the TSH receptor and cyclic AMP signaling pathway. Adequate radioactive iodine uptake therefore requires elevation of serum TSH, usually to a target above 30 mIU/L, which is achieved either by the traditional approach of levothyroxine discontinuation or by recombinant TSH administration.

The traditional approach involves discontinuation of levothyroxine for approximately four weeks, allowing endogenous TSH to rise as negative feedback inhibition is removed. An alternative approach may be used by shortening the hypothyroid interval by transitioning patients from levothyroxine to liothyronine (T_3_) for two to four weeks, followed by withdrawal of T_3_ for a further two weeks; T_3_ has a considerably shorter serum half-life than T_4_. This modified protocol reduces the total duration of biochemical hypothyroidism required to achieve an adequate TSH rise [49,50]. Thyroid hormone withdrawal reliably achieves the target TSH threshold but is associated with symptomatic hypothyroidism, including fatigue, cognitive slowing, depressed mood, constipation, and reduced quality of life, which may be particularly poorly tolerated in elderly patients or those with pre-existing cardiovascular or psychiatric comorbidity.

Recombinant human TSH (rhTSH) permits TSH stimulation without inducing hypothyroidism (Table 2). The standard protocol involves intramuscular injection on two consecutive days, with radioactive iodine administered on the third day, while the patient continues levothyroxine replacement throughout. Multiple randomized controlled trials have demonstrated that rhTSH-aided preparation achieves remnant ablation rates equivalent to thyroid hormone withdrawal in low- and intermediate-risk disease, while avoiding the morbidity of induced hypothyroidism [50,51]. Use of rhTSH in patients with known distant metastatic disease or very high-risk disease remains comparatively less validated and more controversial than in the remnant-ablation setting, although off-label use in this population has been increasing as clinical experience accumulates.

Because non-radioactive (stable) iodine competes with ^131^I for uptake via NIS, depleting the body’s iodine pool prior to treatment increases the relative fraction of the administered radioactive dose taken up by target tissue. A low-iodine diet is generally recommended for one to two weeks prior to radioactive iodine administration. A low-iodine diet involves restriction of iodized salt, dairy products, eggs, seafood and other marine products, soy-based foods, dyes and preservatives and multivitamin preparations containing iodine. It is important to avoid radiographic contrast media for 4 to 8 weeks prior to radioiodine treatment, as the iodine load from contrast agents can suppress radioiodine uptake for a prolonged period. Amiodarone has a high iodine content and long half-life; it can suppress iodine uptake by the thyroid for many months following discontinuation of the drug [52]. Therefore, amiodarone use should be reviewed in advance of planned radioiodine treatment.

Pregnancy should be excluded prior to the administration of radioactive iodine in all female patients with childbearing potential by performing a pregnancy test. Breastfeeding should be discontinued, as radioiodine concentrates in lactating breast tissue and presents a radiation risk to the infant. Female patients should avoid pregnancy for at least 6 months after the administration of radioactive iodine. Radioactive iodine should not be administered during breastfeeding and should be deferred until at least 3 months after breastfeeding or pumping has completely stopped. Male patients who have received a cumulative activity above 14.8 GBq (400 mCi) should be counseled on the potential risk of infertility, with a suggested interval of at least 120 days before attempting conception.

A complete blood test should be performed, and renal function should be estimated prior to the administration of radioactive iodine, as impaired renal clearance prolongs whole-body radiation exposure and may necessitate dose adjustment. Sialagogues, namely sour candies or lemon drops, may be administered to stimulate salivary flow and reduce the risk of radiation-induced sialadenitis. They should be administered following radioactive iodine administration. However, later, rather than immediate, administration of sour candies or lemon drops may be preferrable as sialadenitis, dry mouth and permanent xerostomia was found to be more frequent after immediate administration, after one hour, as compared to later administration, after 24 h [53]. Adequate hydration and frequent bladder emptying are encouraged to minimize radiation exposure in the bladder and surrounding anatomic structures.

Pre-treatment diagnostic whole-body scanning with low ^131^I or ^123^I activity is applied in selected cases. In many centers all around the world, post-treatment scanning is applied to minimize the so called “stunning” phenomenon, which may limit subsequent therapeutic uptake [54,55]. Baseline thyroglobulin and TgAb levels should be measured prior to treatment to serve as baseline measurements ensuring future follow-up. Radiation safety counseling should be provided, to ensure health of any nearby children and any pregnant females and hygiene measures should be advised for approximately 3 to 7 days following radioactive iodine administration.

In summary, serum TSH should be greater than 30 mIU/L if thyroid hormone withdrawal was performed or as established in the standard rhTSH protocol, thyroglobulin and TgAb should be measured at baseline, and recent iodinated contrast exposure and amiodarone dose should be reviewed.

## 10. Dedifferentiation in Thyroid Cancer

A percentage of approximately 5–15% of differentiated thyroid carcinomas (DTC) progress to radioiodine refractory disease through the process of dedifferentiation over the course of the disease, thus becoming refractory to the administration of radioactive iodine [17,56,57]. Poorly differentiated thyroid cancer and anaplastic thyroid cancer may harbor more mutations and are dedifferentiated [17,58,59]. Anaplastic thyroid carcinoma may arise from a pre-existing differentiated thyroid carcinoma, in particular papillary carcinoma.

Extensive research has indicated that dedifferentiation of differentiated thyroid carcinoma is a progressive process in which mutations of several genes, including BRAF and RAS, drive progression of cancer cells from differentiated thyroid carcinoma to poorly differentiated thyroid carcinoma, and even anaplastic thyroid cancer, by activation of signaling pathways such as mitogen-activated protein kinase (MAPK) [60]. This process leads to aggressive tumor biological behavior, increasing metastatic potential and resistance to treatment methods, and deteriorating prognosis. According to the ATA 2025 guidelines, absence of uptake on post-therapy scanning in the presence of structurally evident disease, and/or progression within 6 months of an adequate therapeutic administration of radioiodine, in which uptake was confirmed, are characteristics of dedifferentiated thyroid cancer [15]. According to the ATA guidelines, identification of radioactive refractory thyroid cancer may be performed following the administration of therapeutic radioiodine [15].

Loss of NIS membrane trafficking (even when NIS mRNA/protein is still present intracellularly, mislocalized rather than absent), BRAF V^600E^-driven mitogen-activated protein kinase pathway activation, silencing of thyroid differentiation genes, and dedifferentiation in aggressive types of histology (tall cell, poorly differentiated) all reduce iodine avidity [60,61]—this is the rationale behind MEK/BRAF inhibitor “redifferentiation” protocols (e.g., selective use before radioactive iodine to transiently restore NIS expression and re-sensitize refractory tumors).

## 11. Redifferentiating Agents for Thyroid Carcinoma

Dedifferentiation in thyroid cancer is driven primarily by mitogen-activated protein kinase pathway activation, which suppresses expression of iodine-handling machinery—most importantly the sodium-iodide symporter (NIS/SLC5A5), but also thyroglobulin, TSH receptor, and TPO [17]. The degree of tumor dedifferentiation correlates with the magnitude of mitogen-activated protein kinase activation, with BRAF^V600E^ mutations producing greater pathway activation than RAS or RTK alterations [62,63,64]. This gave rise to the therapeutic logic of redifferentiation: pharmacologically suppressing mitogen-activating protein kinase activation signaling to restore NIS expression and radioactive iodine uptake, thereby converting a previously radioactive iodine-refractory tumor back into one that can be treated with radioiodine [65].

Various clinical studies have indicated that targeting the MAPK pathway may restore radioiodine uptake in dedifferentiated thyroid carcinoma patients (Table 3) [66,67,68,69,70,71,72,73,74]. Selumetinib, a MEK inhibitor, showed response in some patients, especially patients with RAS mutations [66,75]. In the SEL-I-METRY trial, a phase II trial, selumetinib 75 mg administered twice per day for a period of 28 days followed by the administration of radioactive iodine treatment led to improved progression-free survival in the group of patients who showed increased iodine (^123^I) uptake [76]. BRAF inhibitors, such as dabrafenib and vemurafenib, were tested in clinical trials [71,77,78,79]. Tumor regression was noted with vemurafenib, suggesting restoration of iodine uptake in conjunction with alternative mechanisms contributing to tumor control [80]. Notably, some radioactive iodine refractory patients experienced tumor regression with vemurafenib monotherapy, suggesting mechanisms beyond restored iodine uptake may contribute to tumor control. As monotherapy may have limited effects, combination treatment methods were introduced. Consequently, combination strategies have become a focus of research. The combination of dabrafenib and trametinib has been successfully applied for the treatment of advanced radioactive iodine refractory thyroid cancer harboring the BRAF^V600E^ [71,81,82]. However, other researchers have not reported beneficial effects of this combination. Jaber et al. reported that effective restoration of radioactive iodine uptake was achieved in both BRAF- and RAS-mutated tumors on targeted therapy [68]. A Phase II multicenter study found no significant difference in objective response rates between dabrafenib monotherapy and dabrafenib trametinib combination therapy [74]. Additionally, results have indicated that the redifferentiating effect may be transient. The selective NTRK inhibitor larotrectinib has also been tested and it induced increased iodine uptake in pulmonary metastases in patients with EML4-NTRK3 fusions [83]. The RET inhibitor selpercatinib has also demonstrated efficacy in papillary thyroid carcinoma, in both adult and pediatric patients, with RET fusions [84]. The application of multikinase inhibitors combined with MAPK inhibitor in simultaneous or sequential administration may also have therapeutic potential.

Redifferentiation was first attempted with agents like romidepsin and sorafenib, but these failed to produce meaningful clinical impacts. Isotretinoin and lithium have also been applied. The field shifted decisively once the application of mitogen-activated protein kinase inhibitors, for a limited period followed by high activity ^131^I, showed promising results in the restoration of iodine uptake, and tumor response approaches emerged [85]. Redifferentiation has been tested in patients with BRAF-mutated and RAS-mutated tumors in various trials. Groussin et al. [86] investigated the redifferentiating effect of larotrectinib in three patients with advanced metastatic NRTK-rearranged radioactive iodine refractory thyroid cancer and they observed a redifferentiating effect and restored radioactive iodine uptake in two patients. The authors noted that administration of larotrectinib may lead to redifferentiation of the advanced thyroid tumor and the possibility of the added therapeutic effect of radioactive iodine. It should be noted that kinase inhibitors themselves have antitumor effects which may also contribute to thyroid tumor treatment. Kinase inhibitors are applied for a limited period of time and then withdrawn as radioactive iodine avidity of the tumor is restored. Thus, the adverse effects of kinase inhibitors are limited.

## 12. Conclusions

Radioactive iodine treatment is applied in patients with follicular-derived differentiated thyroid carcinoma. Remnant ablation after total thyroidectomy is not routinely recommended in low-risk differentiated thyroid carcinoma. Adjuvant radioactive iodine may be considered in both low–intermediate and intermediate–high risk of recurrence differentiated thyroid carcinoma. Radioactive iodine is routinely recommended in high-risk patients and in patients presenting with distant metastatic disease. Effective radioiodine therapy in differentiated thyroid cancer depends on systematic preparation that maximizes TSH-driven radioiodine avidity while minimizing competing stable iodine intake. The choice between thyroid hormone withdrawal and recombinant human TSH stimulation should be individualized based on disease risk category, comorbidities, and patient tolerance of transient hypothyroidism. Attention to dietary iodine restriction, reproductive safety, renal function, and radiation protection protocols further ensures both the efficacy and safety of treatment. Dedifferentiation of thyroid carcinoma limits radioactive iodine avidity. Redifferentiating agents, and in particular, short-term treatment with kinase inhibitors, may restore radioactive iodine avidity and enable the administration of radioactive iodine to dedifferentiated thyroid carcinoma patients.

## Figures and Tables

**Table 1:** Response of differentiated thyroid carcinomas to treatment (RAI = radioactive iodine, Tg = thyroglobulin, TgAb = anti-thyroglobulin antibodies) (Ringel et al. 2025) [15].

Treatment Received	Excellent Response	Indeterminate Response	Biochemically Incomplete Response	Structurally Incomplete Response
Total thyroidectomy + RAI	non-stimulated Tg < 0.2 ng/mL, or stimulated Tg < 1 ng/mL, (-) TgAb, (-) evidence of disease	non-stimulated Tg 0.2–1 ng/mL or stimulated Tg 1–10 ng/mL TgAb stable/declining nonspecific imaging findings	non-stimulated Tg > 1 ng/mL or stimulated Tg > 10 ng/mL or increasing TgAb levels (-) imaging	structural evidence of disease
Total thyroidectomy no RAI	non-stimulated Tg < 2.5 ng/mL (-) TgAb, (-) imaging	non-stimulated Tg 2.5–5 ng/mL TgAb stable/declining nonspecific imaging findings	non-stimulated Tg > 5 ng/mL increasing TgAb levels (-) imaging	structural evidence of disease
Lobectomy	normal contralateral lobe, (-) lymph nodes			structural evidence of disease

**Table 2:** Methods of preparation for radioactive iodine (RAI) administration in thyroid cancer.

Parameter	Thyroid Hormone Withdrawal (THW)	Recombinant Human TSH (rhTSH)
Method	Levothyroxine stopped for ~4 weeks, or T3 substitution for 2–4 weeks then withdrawn for 2 weeks	Two consecutive intramuscular injections; RAI given on day 3
Euthyroid status	Hypothyroidism	Euthyroidism
Quality of life	Impaired	Preserved
Efficacy	Established	Non-inferior
Use in high-risk/metastases	Traditionally preferred	Less validated
Preferred	If maximal TSH elevation is prioritized	In comorbidities

**Table 3:** Clinical trials of agents applied therapeutically for redifferentiation in dedifferentiated thyroid cancer patients.

Clinical Trial	Pharmaceutical Agents and Targeted Mutation)	Method of Restored Uptake Definition	Dosimetry/I-131 Activity
Ho 2013 [66]	Selumetinib (MEK)	I-124 PET after thyrotropin alfa stimulation, before and 4 weeks after selumetinib	I-131 given if second I-124 PET showed a lesion dose of at least 2000 cGy was achievable; 8 of 12 patients with restored uptake reached the threshold.
Rothenberg 2015 [67]	Dabrafenib (BRAF)	Diagnostic I-131 whole-body scan after rhTSH; new uptake sites in 6 of 10 patients	No dosimetry; fixed empiric 5.5 GBq (about 150 mCi)
Jaber 2018 (retrospective) [68]	Dabrafenib, vemurafenib, trametinib or investigational MEKi	Diagnostic I-131 whole-body scan during treatment; median TKI duration before scan at 14.4 months	Median 7.4 GBq; only 3 patients had pre-therapy dosimetry, the rest empiric activity
Dunn 2019 [73]	Vemurafenib (BRAF)	Pre/post I-124 PET with rhTSH; new or increased uptake in 6 of 10	Treated if at least one previously negative lesion of 5 mm or more could receive 20 Gy or more with 11.1 GBq or less; 4 of 6 qualified
Weber 2022 (ERRITI) [72]	Trametinib (BRAF-WT) or trametinib + dabrafenib (BRAF-MUT), 21 ± 3 days	Post-treatment I-123 SPECT/CT read visually: target-to-background ratio above 4 and uptake at least 2x mean liver uptake in at least one lesion	Personalized I-131 activity from lung, blood and lesion dosimetry (I-124 PET); mean about 300 mCi
Leboulleux 2023 [71] (dabrafenib + trametinib)	Dabrafenib + trametinib, 42 days	Radioiodine scan on day 28; post-therapy scan showed uptake in 20 patients; scan did not gate treatment	Fixed empiric 5.5 GBq after rhTSH regardless of diagnostic scan result
Leboulleux 2023 [70] (trametinib, RAS; MERAIODE)	Trametinib, 42 days	Radioiodine scan on day 28 of the course	Fixed 5.5 GBq on day 35 (±2) regardless of scan result
Iravani 2019 [69]	Trametinib (NRAS) or BRAF + MEK inhibitor combinations (BRAF V600E), 4 weeks	I-124 PET at two time points; 4 of 6 patients judged suitable for radioiodine on I-124 PET results	Absorbed dose estimated from two-time-point I-124 imaging using OLINDA sphere-model factors; added to decision-making as the protocol evolved
Busaidy 2022 [74]	Dabrafenib +/− trametinib	Not known	Not known

## Data Availability

No new data were created or analyzed in this study. Data sharing is not applicable to this article.
